# West Nile Virus Temperature Sensitivity and Avian Virulence Are Modulated by NS1-2B Polymorphisms

**DOI:** 10.1371/journal.pntd.0004938

**Published:** 2016-08-22

**Authors:** Elizabeth A. Dietrich, Stanley A. Langevin, Claire Y.-H. Huang, Payal D. Maharaj, Mark J. Delorey, Richard A. Bowen, Richard M. Kinney, Aaron C. Brault

**Affiliations:** 1 Division of Vector-Borne Diseases, Centers for Disease Control and Prevention, Fort Collins, Colorado, United States of America; 2 Department of Pathology, Microbiology and Immunology, School of Veterinary Medicine, University of California, Davis, Davis, California, United States of America; 3 Department of Biomedical Sciences, Colorado State University, Fort Collins, Colorado, United States of America; University of Texas Medical Branch, UNITED STATES

## Abstract

West Nile virus (WNV) replicates in a wide variety of avian species, which serve as reservoir and amplification hosts. WNV strains isolated in North America, such as the prototype strain NY99, elicit a highly pathogenic response in certain avian species, notably American crows (AMCRs; *Corvus brachyrhynchos*). In contrast, a closely related strain, KN3829, isolated in Kenya, exhibits a low viremic response with limited mortality in AMCRs. Previous work has associated the difference in pathogenicity primarily with a single amino acid mutation at position 249 in the helicase domain of the NS3 protein. The NY99 strain encodes a proline residue at this position, while KN3829 encodes a threonine. Introduction of an NS3-T249P mutation in the KN3829 genetic background significantly increased virulence and mortality; however, peak viremia and mortality were lower than those of NY99. In order to elucidate the viral genetic basis for phenotype variations exclusive of the NS3-249 polymorphism, chimeric NY99/KN3829 viruses were created. We show herein that differences in the NS1-2B region contribute to avian pathogenicity in a manner that is independent of and additive with the NS3-249 mutation. Additionally, NS1-2B residues were found to alter temperature sensitivity when grown in avian cells.

## Author Summary

West Nile virus (WNV) is a mosquito-borne virus that has caused outbreaks in humans in many regions of the world. Birds are the natural hosts for WNV. However, different strains of WNV cause different disease outcomes in birds. Here, we compared two WNV strains, one of which causes higher mortality and generates more virus in American crows than the other. Previous research has shown that this difference is due in large part to a difference between the two strains at a single amino acid in the NS3 gene; however, this difference does not completely explain the observed effect. Here we show that another region of the viral genome also affects disease outcomes in American crows, and changes the sensitivity of the virus to temperature when grown in bird cells. These findings help us to understand the genetic features that affect WNV infection and disease outcomes in its natural host. Detection of such features in new strains of WNV and related viruses could help to understand and predict future outbreaks.

## Introduction

West Nile virus (WNV) is the most widely distributed flavivirus in the world, occurring on all continents except Antarctica [1,2]. Recent human disease outbreaks in Europe and North America have brought increased scientific and public health attention to WNV; however, WNV may also cause significant underreported disease in developing countries [1,3–8]. Despite some advances, significant gaps remain in our knowledge of the ecological and genetic determinants of WNV transmission and disease.

WNV is maintained in avian reservoir hosts and is transmitted by *Culex spp*. mosquitoes [9,10]. Infection rates of mosquito vectors with WNV are proportionate to the virus titer in the infectious blood meal, with host sources generating titers below approximately 10^5^ plaque-forming units (pfu)/ml sera considered to be poorly infectious to mosquitoes [11–14]. In contrast, birds of the family *Passeridae* can develop very high viremia titers, up to approximately 10^10^ pfu/ml in some corvids, and are considered to be the most relevant reservoir hosts that drive the force of epizootic/epidemic transmission [15–17].

For maximum transmissibility, WNV strains must be able to replicate at a variety of temperatures, from approximately 14°C external temperatures experienced by mosquitoes to 45°C body temperatures of febrile avian hosts [18–22]. Strains that cannot withstand the high temperatures experienced by febrile birds are expected to be at a competitive disadvantage for viremogenesis and subsequent transmission [18,23]. Indeed, flavivirus strains and mutants that are temperature sensitive (ts) *in vitro* are frequently also attenuated *in vivo* [23–27].

WNV, like other members of the *Flavivirus* genus, encodes a polyprotein that is post-translationally processed into three structural proteins (the capsid protein C and envelope proteins prM and E) and seven nonstructural proteins (NS1, NS2A, NS2B, NS3, NS4A, NS4B, and NS5). WNV is phylogenetically divided into at least five lineages, with the majority of circulating and epidemic strains belonging to lineage 1 [2]. WNV was isolated in the Americas for the first time in New York in 1999, and rapidly spread across the continent [28]. The NY99 strain, representative of the East Coast genotype of lineage 1a WNVs, has been extensively studied and is widely used as a model strain for WNV studies. The current strains circulating in North America represent a different genotype that is derived from the NY99 ancestor [29]. An alternative lineage 1a WNV strain that was isolated in Kenya, KN3829, shares a high genetic identity with NY99 with a total of 11 amino acid differences between the two strains (Table 1) (Genbank: AF196835 [NY99] and AY262283 [KN3829]).

**Table 1:** Genetic differences between WNV NY99 and KN3829.

Gene	Position	NY99	KN3829
C	108	K	N
C	113	V	A
E	126	I	T
E	159	V	I
NS1	70	A	S
NS2A	52	T	A
NS2B	103	V	A
NS3	249	P	T
NS3	356	T	I
NS4A	85	A	V
NS4B	249	E	D
3′ UTR	22 nucleotide differences		

American crows (AMCRs; *Corvus brachyrhynchos*) infected with North American strains of WNV exhibit high levels of mortality and high viremia titers [15,30–32]. In laboratory infection studies, WNV NY99 typically elicits a viremia of over 10^8^ plaque forming units (pfu)/ml sera, and 100% mortality within approximately 6–7 days [15,18,26,30,33]. Due to their high susceptibility and visibility, AMCRs have been used as a sentinel species for WNV circulation in North America [34]. Despite the high genetic relatedness with NY99, KN3829 exhibits a strikingly different avian virulence phenotype, eliciting very low viremia and limited mortality in AMCRs [18,30,33]. Previous research has demonstrated that a single, positively-selected amino acid substitution at residue 249 in the NS3 helicase gene of WNV is strongly associated with virulence in AMCRs [30]. The NY99 strain encodes a proline residue at this position, while KN3829 encodes a threonine residue (Table 1). Introduction of an NS3-P249T mutation into the NY99 backbone reduced AMCR viremia by almost 10^6^-fold, while the reciprocal mutation in the KN3829 virus (KN3829-NS3-T249P) increased viremia to a similar degree [26,30].

However, a residual difference in virulence between the two strains was not attributable to the NS3-249 amino acid difference. KN3829 and KN3829-NS3-T249P elicited approximately 10-fold lower viremia than NY99-NS3-P249T and NY99, respectively [30]. Mortality was also reproducibly lower with the NS3-249T mutant virus created in the NY99 backbone. Therefore, we hypothesized that other amino acid polymorphisms, or differences in the 3′ untranslated region (UTR), could account for this difference in pathogenesis and/or be associated with stabilization of the KN3829 virus. To test this hypothesis, we generated chimeric virus constructs between the infectious clones of NY99 and KN3829, and used the resulting viruses for evaluation of pathogenic potential in AMCRs and growth at standard and elevated temperatures in avian cell culture.

## Materials and Methods

### Construction of chimeric and mutant virus constructs

**Fig 1:**
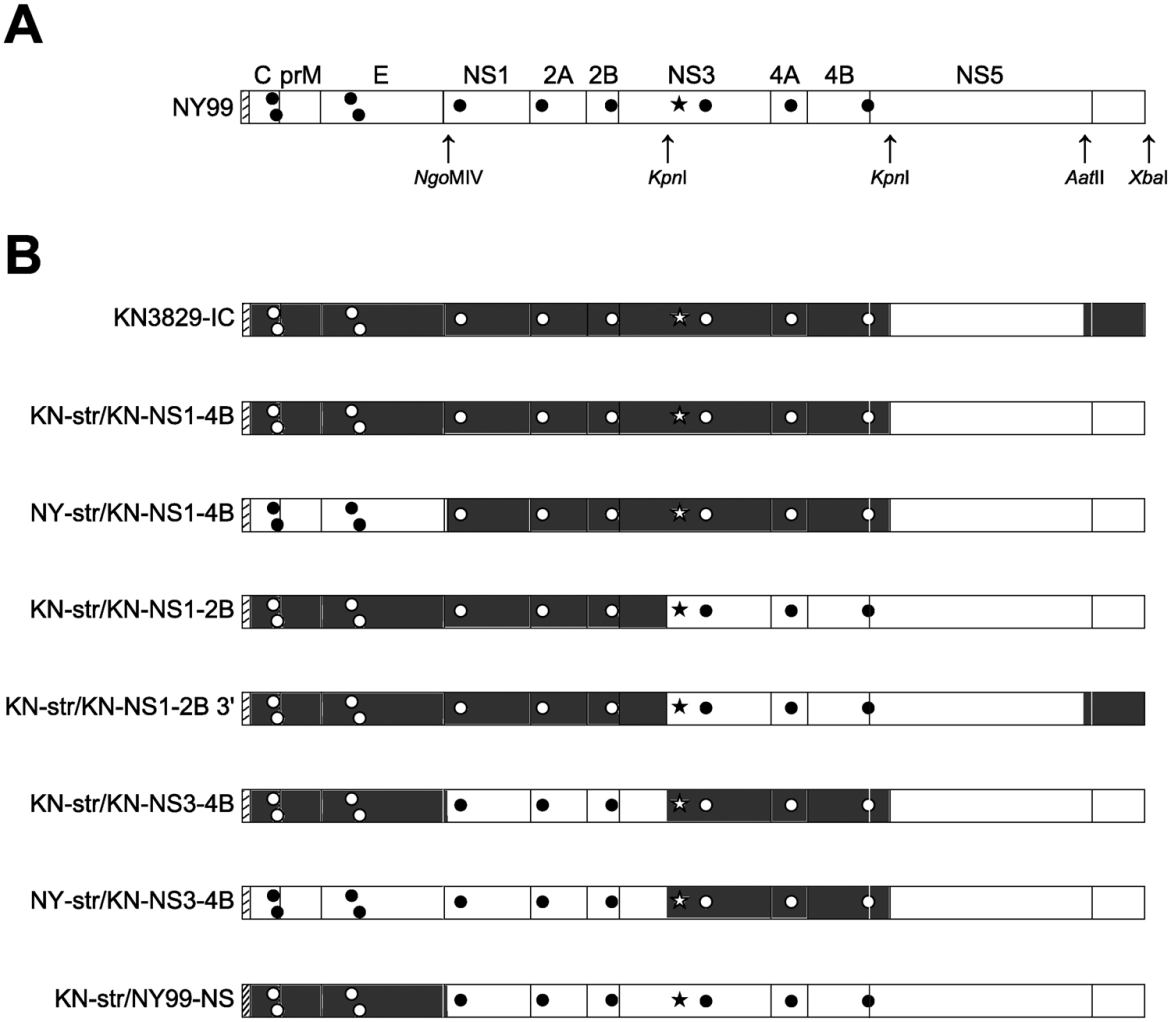
Construction of NY99/KN3829 chimeras. (A) Diagram of WNV NY99 genome structure showing the location of nonsynonymous differences from KN3829 (Table 1) and restriction enzyme sites used to create chimeras. Star, NS3-249. (B) Chimeric viruses used in this study. Black symbols on white background, NY99. White symbols on black background, KN3829. The 5′ UTR is identical between the two isolates (indicated with a striped background). The amino acid sequence of NS5 is also identical between the isolates; however, there are synonymous differences in this gene. The NS5 of the NY99 strain was used in all constructs, for convenience in cloning. NS3-T249P mutants in KN3829-IC, KN-str/KN-NS1-4B, and NY-str/KN-NS3-4B were also created and indicated in strain designations with (Pro).

Infectious clones of NY99 and KN3829 were described previously [18]. To create chimeric constructs, we divided the viral genome into segments based on conveniently located restriction sites: *Ngo*MIV at nucleotide (nt) 2495 (in NS1; used for ligation of the two-plasmid system during virus rescue); *Kpn*I at nt 5341 (in NS3); *Kpn*I at nt 7762 (beginning of NS5); and *Aat*II at nt 10203 (end of NS5). Segments from the wild-type KN3829 and NY99 infectious clones, as well as the KN3829-NS3-T249P mutant virus, were interchanged using these restriction sites. Chimeric virus strains were named based on the KN3829-specific genome segments they contained (Fig 1). NS1-2B point mutations were created in the KN-IC (CG plasmid) infectious clone by site-directed mutagenesis as previously described [23].

### Virus rescue

Rescue of infectious clone-derived virus was described previously [18]. Briefly, the 5′ and 3′ plasmids of NY99, KN3829, and mutant and chimeric viruses were digested with *Ngo*MIV, ligated, and linearized with *Xba*I (New England Biolabs) before *in vitro* transcription with the Ampliscribe High-Yield T7 Transcription kit (Epicentre Biotechnologies). Viral RNA was transfected into BHK-21 cells by electroporation. When >50% of cells displayed cytopathic effect, supernatant was harvested, centrifuged to remove cellular debris, and stored at -70°C until titration by plaque assay. RNA was extracted from stocks from individual clone-derived viruses and viral genomes were sequenced as described previously [26].

### Cell culture and growth kinetics

Vero, BHK-21, and duck embryonic fibroblast (DEF) cells were maintained in DMEM containing 10% FBS, 100 U/ml penicillin, and 50 μg/ml streptomycin. For determination of growth kinetics, DEF cells were inoculated with virus at an MOI of 0.1. After a one hour adsorption at 37°C, cells were washed three times with Dulbecco’s PBS (Life Technologies), growth medium was replaced, and cells were placed in incubators at either 37° or 44°C. Supernatant was sampled daily for five days. 30 μl of each sample was added to 270 μl of fresh medium containing 20% FBS, frozen, and stored for titration as above.

AMCR peripheral blood mononuclear cells (PBMCs) were isolated using Histopaque-1077 (Sigma-Aldrich) and maintained in RPMI containing 10% FBS, penicillin/streptomycin as above, and 1 μg/ml Fungizone (Life Technologies), as described previously [35]. PBMCs were inoculated with virus at an MOI of 10, incubated for one hour at 37°C or 42°C, and then centrifuged at 1500×g, resuspended in fresh growth medium, and incubated at the same temperature. One third of the well volume was sampled and replaced daily for six days, and samples were stored as described above.

### Animal studies

After-hatch year AMCRs were trapped using cannon nets in Bellvue, Colorado between 2004–2007. Crows were banded, bled, and tested for pre-existing immunity to WNV and St. Louis encephalitis virus using plaque reduction neutralization tests as previously described [33]. AMCRs were housed at Colorado State University in groups of 2–3 in 1-m^3^ cages and fed an *ad libitum* mixture of dry dog and cat food. Groups of 16 AMCRs were inoculated subcutaneously with 1500 pfu of parental, chimeric, or point mutant WNV in a 100 μl volume. Inoculated AMCRs were bled by jugular venipuncture daily for seven days. Whole blood was diluted 1:10 in DMEM containing 10% FBS and penicillin/streptomycin. Blood samples were allowed to coagulate at room temperature before centrifugation for 10 min at 4000 × g, and were stored at -70°C until titration by plaque assay. AMCRs were monitored daily for 14 days and any birds displaying signs of WNV disease, such as ataxia, incoordination, or difficulty feeding, were euthanized by intravenous phenobarbital overdose. All surviving birds were euthanized at day 14 in the same manner.

### NS3-249 sequence analysis

RNA was extracted from selected samples of PBMC culture supernatant or AMCR blood using a Viral RNA mini kit (Qiagen) as described previously [26]. RT-PCR was performed using a SuperScript III One-Step RT-PCR kit (Life Technologies) and primers WNV5032F (5′-GGAACATCAGGCTCACCAATAGTGG-3′) and WNV5497R (5′-CTTTGTGGAAATGTAACCTCTTGCTGC-3′). The resulting RT-PCR product was sequenced with the same primers.

### Statistical methods

All statistical calculations were performed using GraphPad Prism v. 6.04 or R v3.2.2. Statistical analysis of *in vivo* data was performed by synonymizing groups based on NS1-2B genotype (NY or KN) and NS3-249 genotype (Pro or Thr). Survival curves were compared using a log-rank test. Viremia was regressed on dpi assuming polynomial trend and normal errors. The model includes a fixed effect for each modified region of the two viruses, and a random effect for replicates. Times at which peak viremia occurred were estimated from the fit. Standard errors for differences in peak viremia were computed using the delta method and incorporate uncertainty from estimating both time of peak viremia and value of peak viremia. Results were adjusted to account for multiple comparisons, achieving an overall Type I error rate of 0.05.

For temperature-sensitivity data in DEF cells, a semiparametric, mixed model was fit to the titer data. The model includes a fixed effect for each modified region of the two viruses, a random effect for replicates, and temperature-specific mean titer curves. The temperature-specific components were characterized by second degree penalized splines with truncated power basis. The solution to the fit and estimated variances were obtained by computing the best, linear, unbiased predictors of the penalized spline’s representation as a linear, mixed model [36]. Times at which peak titer occurred were estimated from the fit. Standard errors for differences in peak titer were computed using the delta method and incorporate uncertainty from estimating both time of peak titer and value of peak titer. Results were adjusted to account for multiple comparisons, achieving an overall Type I error rate of 0.05.

### Ethics statement

Trapping of AMCRs was performed under US Fish and Wildlife Scientific Collecting Permit MB-032526 and MB-082812. Birds were collected under US Fish and Wildlife Services and Colorado Parks and Wildlife permits with permission of private land owners as well as the managers of the Colorado State Fisheries Unit in Bellvue, CO. Field studies did not involve endangered or protected species. All animal studies presented herein were approved by Institutional Animal Care and Use Committees at the University of California, Davis (approval number 12874) and Colorado State University (approval number 10-2078A). All protocols and practices for the handling and manipulation of crows were in accordance with the guidelines of the American Veterinary Medical Association (AVMA) for humane treatment of laboratory animals as well as the ‘‘Guidelines to the Use of Wild Birds in Research” published by the ornithological council 3rd edition (2010).

## Results

### Residues in the NS1-2B region contribute to virulence in AMCRs

We constructed chimeric virus constructs between NY99 and KN3829 to determine which differences between the two strains, other than the previously described NS3-249 site [26,30], contribute to avian virulence and pathogenesis (Table 1; Fig 1). All chimeric viruses could be grown *in vitro* in rodent (BHK-21) and primate (Vero) cell lines to titers comparable to those attained by the wild-type parental infectious clone viruses (at least 7 log_10_ pfu/ml).

**Fig 2:**
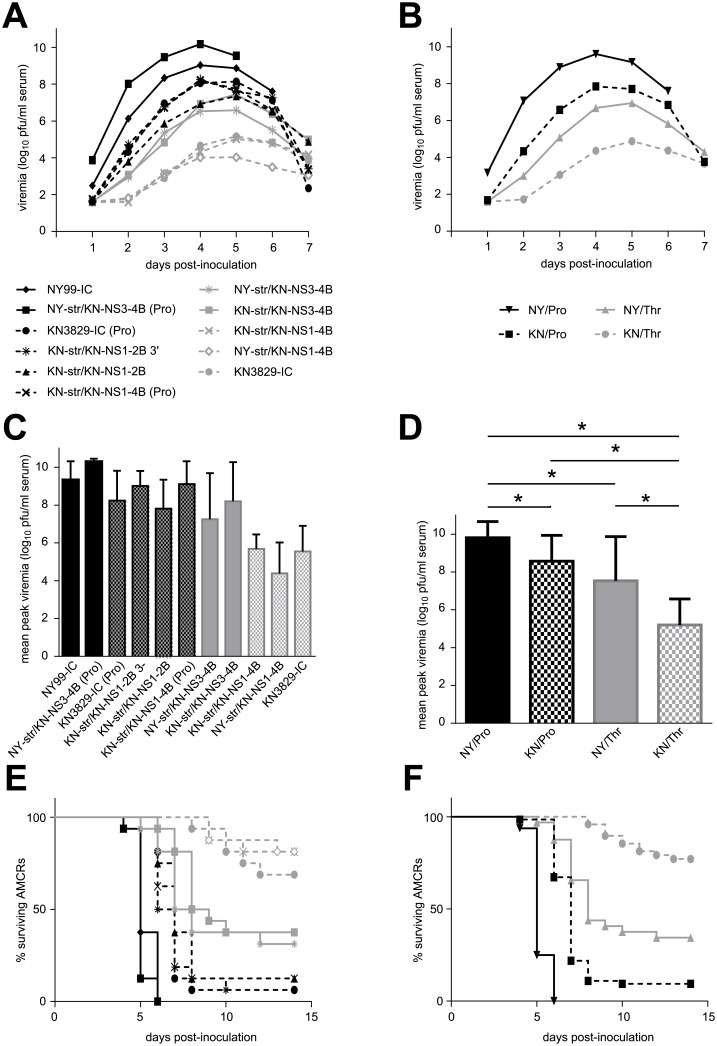
AMCR infections with WNV NY99/KN3829 chimeras. (A) Daily viremia over the first seven dpi. N = 16 AMCRs per group. (B) Daily viremia in groups of birds pooled by NS1-2B genotype (NY, NY99; KN, KN3829) and by NS3-249 genotype (Thr or Pro) All groups are statistically different. (C) Mean peak viremia (+/- standard deviation) in individual groups. (D) Mean peak viremia (+/- standard deviation) in pooled groups. Statistically significant differences are indicated by asterisks. (E) Mortality in individual groups. Symbols are as in (A). N = 8 AMCRs per group. (F) Mortality in pooled groups. Symbols are as in (B). All groups are statistically different from each other (p < 0.001).

AMCRs were inoculated with virus derived from infectious clones of WNV NY99, KN3829, or chimeric plasmids with proline or threonine residues present at the NS3-249 locus. As described previously [18,26,30,33], viremia in NY99-inoculated AMCRs peaked at approximately 10^9^ pfu/ml serum, while KN3829 elicited only approximately 10^5^ pfu/ml serum (Fig 2A). NY99 infection induced 100% mortality within six days post-infection (dpi), whereas 13/16 (81%) of AMCRs infected with clone-derived KN3829 virus survived to 14 dpi (Fig 2E).

As expected, viruses encoding a proline residue at the NS3-249 locus elicited higher viremia (95% CI for difference in peak viremia 0.3 logs to 2.0 logs) (Fig 2A and 2B) and mortality (Fig 2E and 2F) rates than those containing a threonine residue. However, within both Pro and Thr-containing groups, constructs containing the NS1-2B region of NY99 induced statistically higher peak viremia and mortality than those containing the NS1-2B region from KN3829 (Fig 2B and 2F). When groups were synonymized based on genotype at NS1-2B (NY or KN) and NS3-249 (P or T), survival distributions were significantly different (p < 0.001) among all groups (Fig 2F). The peak viremia was significantly different between all pairs of groups except KN/Pro and NY/Thr (Fig 2C and 2D). The structural genes of WNV did not have an apparent effect on pathogenesis in AMCRs. Mortality did not differ between strains that were identical with the exception of their structural genes (p > 0.05) (e.g. compare KN-str/KN-NS3-4B and NY-str/KN-NS3-4B). The difference in mean peak viremia titers between these strains was 0.31 logs (95% CI of -0.1 logs to 0.7 logs). Similarly, the 3′ UTR did not have a detectible effect on viremia or mortality (p > 0.05) (i.e. KN-str/KN-NS1-2B and KN-str/KN-NS1-2B 3’).

We hypothesized that, given the importance of the NS3-249 position for viral replication in AMCRs, infection with Thr-containing viruses may have imposed selective pressure, leading to potential mutations at this site. Therefore, viral RNA extracted from sera collected at 4 dpi from AMCRs infected with KN-str/KN-NS3-4B and NY-str/KN-NS3-4B (8 AMCRs each) was spot sequenced. These viruses were chosen because they grew relatively well in AMCRs compared to other Thr-containing constructs. Of the 16 samples sequenced, only three maintained a Thr residue at NS3-249 with no detectable mutations in the viral population. Eight had mutated to contain an alanine residue at this position. One had mutated to an asparagine residue. The other four contained mixed sequences at the locus. Two had a mixture of alanine and threonine, one had a mixture of alanine and proline, and the last sample contained a mixture of alanine, aspartic acid, and threonine. No other mutations were detected in the surrounding NS3 region.

### NS3-249 modulates replication and NS1-2B modulates temperature sensitivity in avian leukocytes

**Fig 3:**
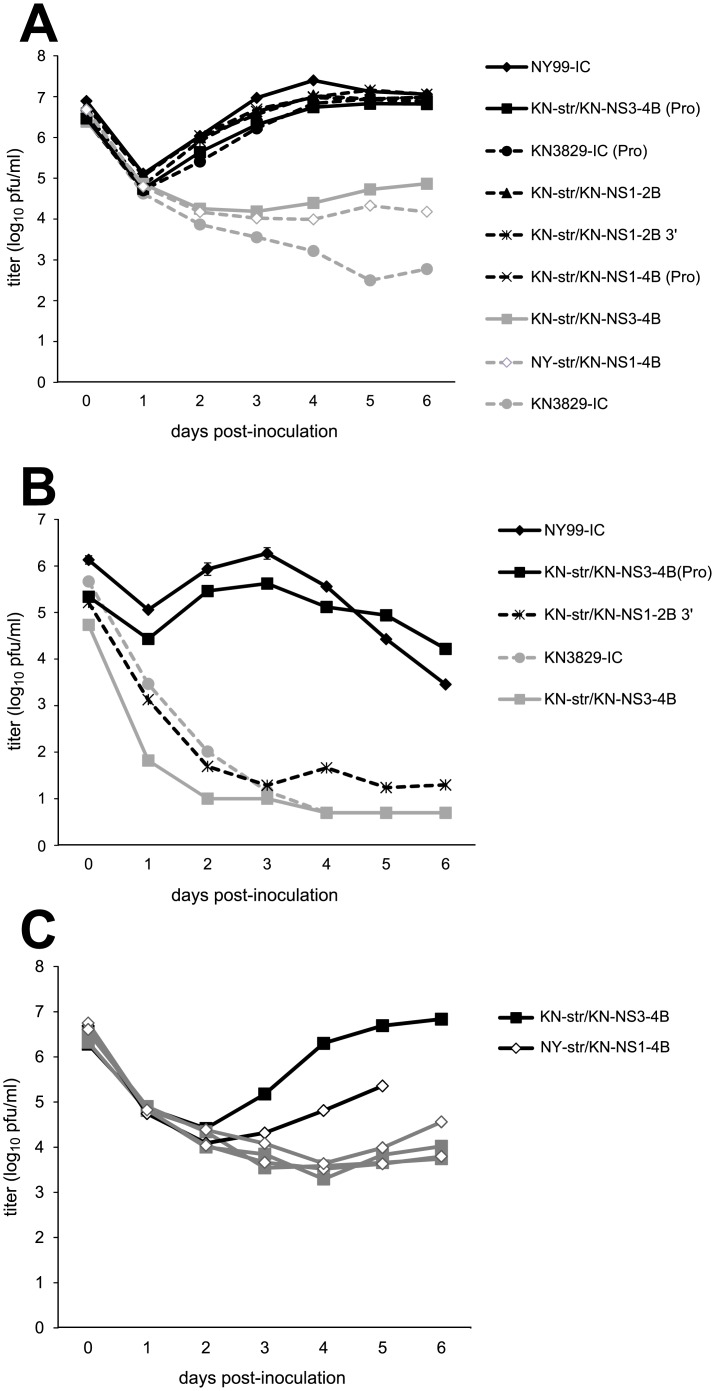
Growth and temperature sensitivity of WNV NY99/KN3829 chimeras in AMCR PBMCs. (A) Growth of chimeric viruses in PBMCs at 37°C. (B) Growth of selected chimeric viruses in PBMCs at 42°C. N = 3 replicates per virus. (C) Variability among replicates in PBMC culture at 37°C. Individual replicates of PBMC cultures of KN-str/KN-NS3-4B and NY-str/KN-NS1-4B are shown. No supernatant was available for one replicate of NY-str/KN-NS1-4B at 6 dpi due to fungal contamination. Filled squares represent KN-str/KN-NS3-4B, and open diamonds represent NY-str/KN-NS1-4B. Viral RNA was extracted from 5 dpi (NY-str/KN-NS1-4B) or 6 dpi (KN-str/KN-NS3-4B) supernatants of cultures shown in black, and the NS3-249 region was sequenced. Cultures shown in grey were not sequenced.

To further analyze the effects of the differences between NY99 and KN3829, AMCR PBMCs were inoculated with the chimeric virus constructs. As described previously [35], replication in AMCR PBMCs at 37°C correlated with NS3-249 genotype (Fig 3A). However, when the temperature was increased to 42°C, the body temperature of AMCRs, only viruses containing both a proline residue and the NS1-2B region from NY99 were able to replicate (Fig 3B).

High variability was observed among the three replicate infections with the KN-str/KN-NS3-4B and NY-str/KN-NS1-4B viruses grown at 37°C. One replicate of the KN-str/KN-NS3-4B culture attained a final titer that was over 100-fold higher than the titers attained by the other two replicates (Fig 3C). Similarly, one replicate of the NY-str/KN-NS1-4B culture attained a titer at 5 dpi that was over 10-fold higher than the other two cultures. Viral RNA was isolated from these two culture supernatants and the region surrounding the NS3-249 region was sequenced [35]. No mutations were found in the NS3-249 residue. However, mutations were found in nearby residues in single high-titered replicates. Specifically, a NY-str/KN-NS1-4B sample contained a mixed population of wild-type and NS3-E251K mutant virus, while a KN-str/KN-NS3-4B sample contained a mixed population of wild-type and NS3-T246I mutant virus. The proximity of these mutations to the NS3-249 site, which modulates PBMC replication, suggests that they may be the cause of the improved growth in these replicates.

#### Multiple genomic regions modulate temperature sensitivity in duck cell culture

**Fig 4:**
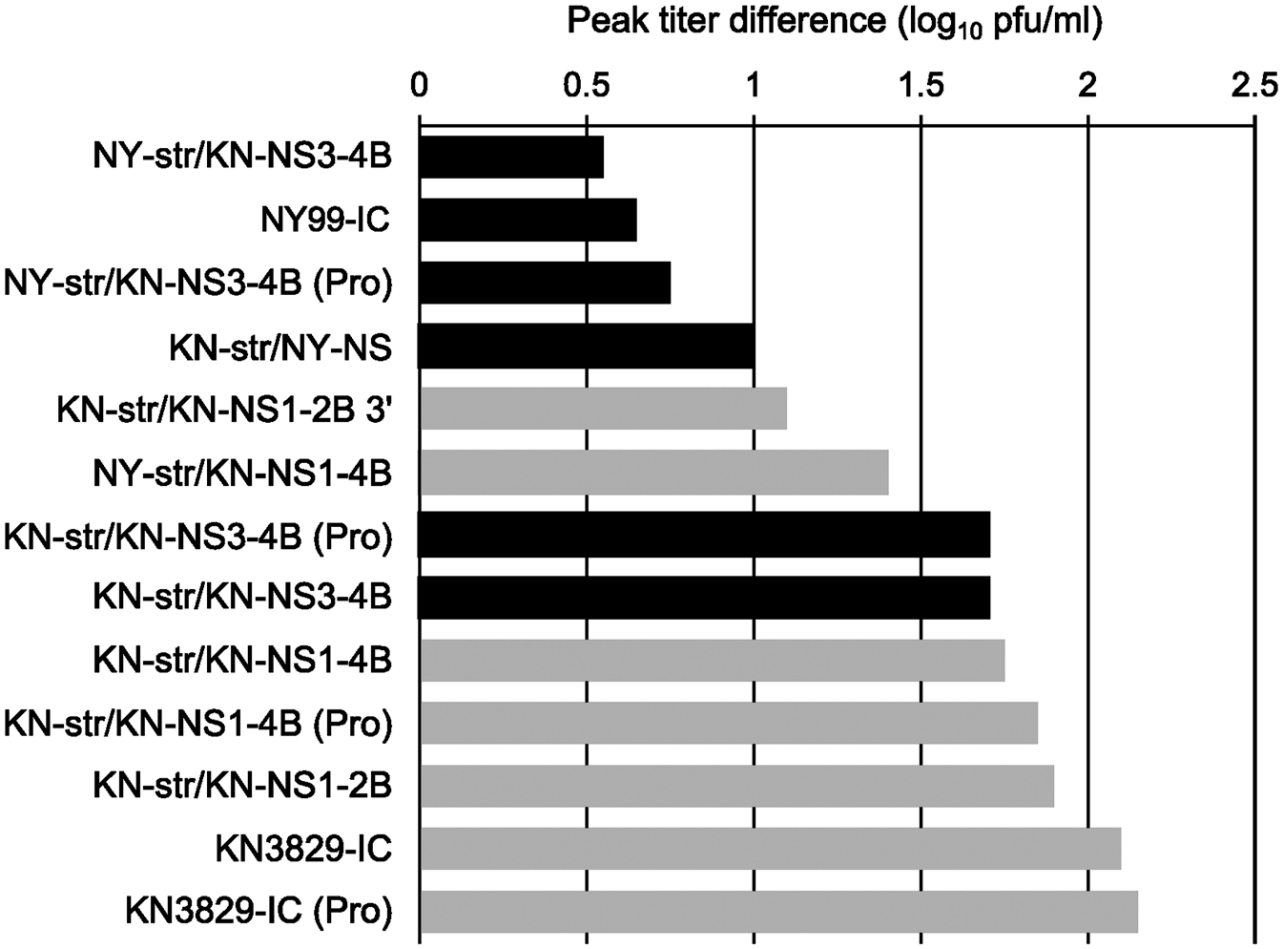
Temperature sensitivity of WNV NY99/KN3829 chimeras in duck embryonic fibroblasts. Chimeric viruses were grown in duck embryonic fibroblasts (DEF) at 37°C and 44°C. The peak titer at 44°C was subtracted from the peak titer at 37°C and plotted in order of increasing temperature sensitivity. Original data are shown in S1 Fig. Black bars, NS1-2B from NY99. Grey bars, NS1-2B from KN3829.

The growth kinetics of WNV chimeric constructs were also examined in duck embryonic fibroblast (DEF) cells (Fig 4; S1 Fig). In contrast with PBMCs, all constructs were capable of replicating in DEF cells at both temperatures. The temperature sensitivity of each isolate was defined for this analysis as the difference in peak titer between growth at 37°C and 44°C. The difference in temperature sensitivity between viruses containing NY99 genes and those containing KN2839 genes at each region was calculated. This analysis found that, on average, chimeras with KN3829-derived genes in the structural, NS1-2B, and NS3-4B (excluding NS3-249) regions are statistically more sensitive to temperature than their counterparts with NY99-derived genes, while differences in the NS3-249 point mutation and the 3′ UTR did not affect temperature sensitivity.

### No single amino acid in NS1-2B modulates virulence in AMCRs

**Fig 5:**
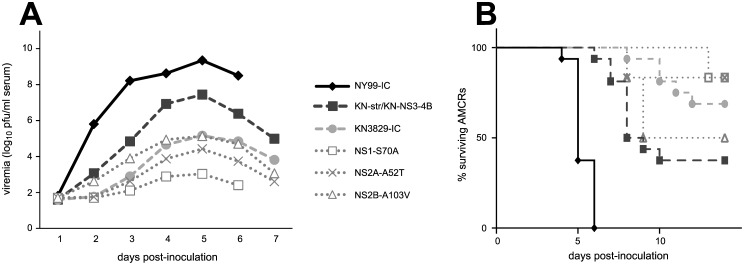
Infection of AMCRs with KN3829 NS1-2B point mutants. Mean daily viremia (A) and mortality (B) in AMCRs infected with viruses containing NS1-S70A, NS2A-A52T, and NS2B-A103V point mutations in the KN3829 backbone. N = 6 AMCRs per group. Data from NY99-IC, KN3829-IC, and KN-str/KN-NS3-4B are shown for comparison (Fig 2).

There are three amino acid differences between NY99 and KN3829 in the NS1-2B region (Fig 1). In order to determine the relative contribution of these three differences to the observed changes in AMCR virulence, we created single NS1-S70A, NS2A-A52T, and NS2B-A103V point mutants in the KN3829 infectious clone backbone. Inoculation of AMCRs with these point mutants led to viremia and mortality that were not distinguishable from wild-type KN3829 (Fig 5).

## Discussion

We show here that replication and virulence of WNV in American crows are modulated by the NS1-2B region of the genome in addition to the previously described effect of the NS3-249 residue. The effects of these two genomic regions are independent and additive *in vivo*. Although the effect of the NS1-2B region is relatively subtle (approximately 10-fold) compared with the effect of NS3-249, it is reproducible and statistically significant. This finding underscores the importance of the flaviviral nonstructural proteins for virulence and viral replication in the natural reservoir host.

As previously described, the NS3-249 site evidently modulates replication in leukocytes [35]. Viral constructs containing a threonine at this position consistently failed to replicate in PBMC culture, while those containing a proline replicated well. No other determinants detectably affected growth in PBMCs at 37°C. However, constructs containing the NS1-2B region from KN3829 were unable to replicate at 42°C. Thus, we conclude that the NS3-249 residue is a determinant of replication in AMCR PBMCs, while the NS1-2B region is a determinant of temperature sensitivity. As febrile AMCRs typically attain body temperatures above 42°C [18], this suggests a possible explanation for the decreased *in vivo* viremia observed in AMCRs infected with these constructs. The combination of leukocyte replication and temperature sensitivity effects may explain the relative *in vivo* virulence of the various constructs.

These results suggest that temperature sensitivity may play an important role in WNV pathogenesis in birds. Interestingly, in North American WNV strains, an NS1-K110N mutation, in combination with a mutation in NS4A, has been associated with *in vitro* temperature sensitivity in DEF cells [23]. Although these findings are not directly comparable to those shown here, they also point to a potential role for NS1 in mediating temperature sensitivity in WNV.

Interestingly, temperature sensitivity of WNV in DEF cells was not directly correlated with temperature sensitivity in AMCR PBMCs. Although previous work found a slight effect of the residue at position NS3-249 on temperature sensitivity in DEF cells in the NY99 genetic background, this effect was not evident in the KN3829 and chimeric genetic backbones assessed here [26]. Instead, the structural genes, NS1-2B region, and NS3-4B region exclusive of NS3-249 all appeared to modulate the differences in DEF cell temperature sensitivity between NY99 and KN3829. This is consistent with chemical mutagenesis studies in dengue virus, in which temperature sensitivity was conferred by mutations in a variety of positions throughout both the structural and nonstructural regions [37]. Temperature sensitivity of flaviviruses is evidently a complex phenotype that can be conferred by a variety of mutations, likely with different underlying mechanisms. These mechanisms may include protein stability and protein-protein interactions, among others. The difference between AMCR PBMCs and DEF cells also indicates that temperature sensitivity results may be dependent on the system used for testing. Although the use of DEF cells for temperature sensitivity testing is convenient, AMCR PBMCs are likely more phenotypically relevant.

None of the three individual amino acid differences in the NS1-2B region between NY99 and KN3829 could individually explain the effect of the overall region on temperature sensitivity or *in vivo* virulence. Thus, the overall effect of this region evidently requires two or more of the amino acid differences. This is consistent with previous results in a dengue-2 vaccine virus study, which showed that a combination of mutations at NS1-53 and NS3-250 was required to make the vaccine virus fully temperature sensitive [38]. The single NS3-250 substitution did not increase temperature sensitivity of the dengue vaccine virus, while the NS1-53 substitution alone only caused subtle temperature sensitivity. Future studies will be required to fully understand the effects of these NS proteins on pathogenesis and temperature sensitivity.

Alternatively, the synonymous nucleotide changes in the NS1-2B region could have an effect at the RNA level, which would not be captured by amino acid point mutations. An RNA secondary structure motif is required for the production of the frameshifted NS1′ protein in WNV and closely related flaviviruses [39]. Mutations that alter this secondary structure can change the ratio of full-length to frameshifted polyprotein, affecting WNV pathogenesis in mice and house sparrows [40,41]. Both NY99 and KN3829 encode the frameshift motif and would be expected to produce NS1′. There are two amino acid differences between the NS1′ coding sequences of NY99 and KN3829, one of which is encoded by the same nucleotide polymorphism that encodes the tested NS2A-A52T mutation. The role of the amino acid sequence of NS1′ is not well understood, and it is possible that the polymorphism not tested here could play a role in pathogenesis and temperature sensitivity. Other cryptic RNA motifs that have not yet been described could also play a role.

The functions of the flaviviral NS1, NS2A, and NS2B proteins are not fully understood, making it difficult to determine why these proteins apparently affect temperature sensitivity in PBMC culture and replication and virulence *in vivo*. The NS1 and NS2A proteins, in particular, have apparent roles in immunomodulation and immunopathogenesis, in addition to their roles in viral replication [42–47]. A silent mutation in WNV-Kunjin virus NS2A that affects the NS1′ frameshift motif also has been shown to alter interferon induction, and an amino acid change at the same position affects apoptosis *in vitro* and virulence in mice [41,48]. Given that the differences in avian pathogenesis observed here appear to be modulated at least in part by replication in immune cells, these immunomodulatory functions may be relevant. Further research on these nonstructural proteins will aid in understanding their role in temperature sensitivity and avian virulence.

Subtle effects of differences among viral strains could have an amplified effect on a larger scale. Although the addition of the NY99 NS1-2B region to virus backbones containing the NS3-249-Thr residue only increased peak viremia titers by approximately 100-fold, AMCRs infected with these viruses experienced viremia titers above 10^5^ pfu/ml for 3–4 days. In contrast, AMCRs infected with the corresponding strains containing the KN3829 NS1-2B region experienced 0–1 days of viremia above 10^5^ pfu/ml. As 10^5^ pfu/ml is the approximate titer required for infection of mosquitoes, this relatively subtle difference could lead to an increased chance of transmission to a mosquito [11–14]. Furthermore, if these determinants in NS1-2B are present in non-North American or alternative lineage WNV strains, increased viremia titers could weaken the potential selective pressure for development of NS3-249P mutations.

These observations highlight the importance of understanding of the determinants of WNV replication and pathogenesis in relevant avian reservoir hosts, including the AMCR. Unraveling the viral genetic factors influencing the infection of different avian species will provide insight into emergence mechanisms of WNV and related flaviviruses. This behavior cannot be predicted based on studies of mammals such as mice, which exhibit physiological, immunological and cytological differences from birds that preclude use as a relevant model system for the selective pressure these viruses undergo during enzootic/epizootic transmission cycles.

## Supporting Information

S1 Fig Temperature sensitivity of WNV NY99/KN3829 chimeras in duck embryonic fibroblasts. Growth of chimeric viruses containing the NS1-2B region from NY99 (A, C, and E) or KN3829 (B, D, and F) at 37°C (A and B) or 42°C (C and D). (E and F), difference in titer at each time point between 37°C and 42°C.
(EPS)
